# Detection and molecular characterization of virulent Newcastle disease virus (subgenotype VII.2) in broiler chickens in Northern Vietnam

**DOI:** 10.14202/vetworld.2023.2086-2095

**Published:** 2023-10-14

**Authors:** Thi Huong Giang Tran, Van Hieu Dong, Van Truong Le, Thi Ngoc Vu, Huu Anh Dang, Thi My Le Huynh

**Affiliations:** 1 Department of Veterinary Microbiology and Infectious Diseases, Faculty of Veterinary Medicine, Vietnam National University of Agriculture, Hanoi, Vietnam; 2 Department of Veterinary Public Health, Faculty of Veterinary Medicine, Vietnam National University of Agriculture, Hanoi, Vietnam

**Keywords:** genetic characterization, Newcastle disease virus, Vietnam, genotype VII.2, virulent strain

## Abstract

**Background and Aim:**

Newcastle disease (ND) is a major viral disease of poultry worldwide. However, data on the molecular characterization of Newcastle disease virus (NDV) in Vietnam are limited. This study aimed to identify the molecular characteristics of NDV strains from the vaccinated chickens farmed in Northern Vietnam.

**Materials and Methods:**

We used reverse-transcription polymerase chain reaction (PCR), sequencing and phylogenetic analysis to characterize NDV strains from vaccinated chicken farms in Northern Vietnam.

**Results:**

Seven out of 72 (9.7%) chicken tissue samples collected from seven chicken farms in the four cities/provinces in northern Vietnam were positive for the NDV genome by PCR method. The complete sequences of the fusion (F) and hemagglutinin-neuraminidase (HN) genes of NDVs isolated in the North of Vietnam from 2021 to 2022 were further evaluated. The results indicated that all seven Vietnamese isolates obtained were reported as virulent NDV strains with the amino acid (AA) sequence of the F0 protein proteolytic cleavage site motif (^112^RRRKRF^117^). Phylogenetic analysis revealed that they were grouped with other NDV class II from subgenotype VII.2, including the two previous Vietnamese NDV (2015), the Chinese (2017), and Southern African (2013) NDV strains. In addition, some AA substitutions were observed in the neutralizing epitopes of the F and HN proteins of the current Vietnamese NDV strains.

**Conclusion:**

The present findings provide useful information for future studies of the evolution of NDVs and improve strategies for ND-controlling programs in Vietnam.

## Introduction

The poultry industry in Vietnam has become a substantial source of animal protein. Such a huge industry is challenged by several devasting pathogens. Newcastle disease (ND) is one of the most economically contagious and fatal viral diseases affecting the poultry industry. Newcastle disease is caused by virulent Newcastle disease virus (NDV) strains. Newcastle disease virus belongs to Avian Orthoavularvirus 1, also called NDV. It is an enveloped negative-sense, single-stranded RNA virus within in the family *Paramyxoviridae*, order *Mononegavirales* [1]. The NDV genome is approximately 15.2 kb in size and encodes six structural proteins such as nucleocapsid protein, phosphoprotein (P), matrix protein (M), fusion protein (F), hemagglutinin-neuraminidase (HN) protein, and large RNA-dependent polymerase (L). In addition, two other V and W proteins could also be coded through P protein mRNA editing [2]. Newcastle disease virus has wide genetic diversity and is divided into class I and II, based on phylogenetic analysis. Viruses from class I belong to a single genotype are avirulent strains, whereas class II viruses are mostly virulent strains classified into 21 genotypes (I-XXI) and many sub-genotypes [1, 2].

At present, genotype VII of class II viruses is responsible for outbreaks in both domestic poultry and wild birds worldwide [3, 4]. Genotype VII viruses are divided into three sub-genotypes (VII.1.1, VII.1.2, and VII.2). Genotype VII.1.1 is considered to have emerged around 1985 in the Far East and rapidly spread to Asia, the Middle East, Europe, and Africa. Genotype VII. 2 viruses caused outbreaks in Indonesia and Malaysia between 2005 and 2010, further spreading to Central and East Asia, the Middle East, Europe, and Africa. They are mostly responsible for the fifth ND panzootic [5–9].

In Vietnam, the first report of an ND outbreak was published in early 1955. At present, ND is endemic in the country, with reports of regular field outbreaks even though vaccination has been largely implemented [10–12]. However, there are few scientific reports on the molecular epidemiology of NDVs circulating in the country. Subgenotypes VIId, VIIh, and XIId were identified from 2002 to 2015 [11–13]. In addition, ND outbreaks are ongoing, there is a need to investigate NDVs causing recent outbreaks in broiler chickens and determine the molecular characterization of the circulating NDV isolates to better control the ND situation better in Vietnam.

This study aimed to detect and identify the molecular characteristics of NDV from broiler chickens with suspected virulent NDV infection in Northern Vietnam.

## Materials and Methods

### Ethical approval

This study does not require ethical approval as per the ethics committee of the institute.

### Study period and location

The study was conducted from September 2021 to November 2022. The samples were collected from four cities/provinces of Northern Vietnam. The study was conducted at Laboratory of Microbiology, Infectious Diseases, Faculty of Veterinary Medicine, Vietnam National University of Agriculture, Vietnam.

### Sample collection

**Figure-1:**
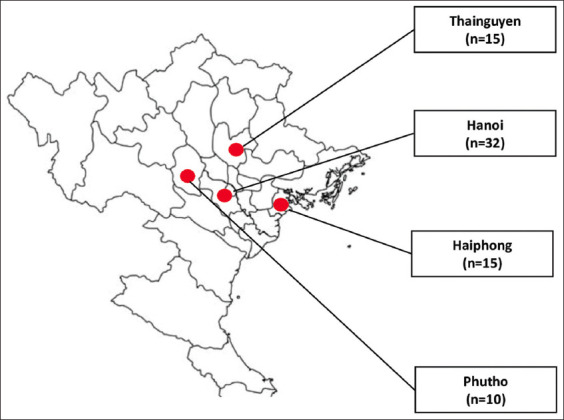
Geographical location of sample areas in northern Vietnam (red circles) and distribution of sampling size in Thainguyen (15), Phutho (10), Haiphong (15), and Hanoi (32). [Source: https://d-maps.com/carte.php?num_car=985&lang=en].

A total of 72 samples from different suspected outbreaks that occurred from 2021 to 2022 were investigated at the Department of Microbiology and Infectious Diseases of Vietnam National University of Agriculture. The samples were collected from seven commercial broiler farms in Thainguyen (TN, n = 15), Haiphong (HP, n = 15), Phutho (PT, n = 10), and Hanoi (HN, n = 32) cities/provinces of Northern Vietnam (Figure-1). All flocks were vaccinated against NDV (La Sota vaccine) with flock size from 1000 to 8000 chickens (Table-1). Sick or dead chickens were submitted to the Department of Microbiology and Infectious Diseases for further analysis. Tracheal swabs or pooled tissues (brain, lung, and spleen) were collected in sterile tubes. A 10% (w/v) tissue homogenate was prepared in phosphate-buffered saline supplemented with gentamicin (10 mg/mL).

**Table-1:** Primers used in this study

Name	Primer sequence (5′–3′)	Fragment size (bp)	Purpose	Reference
APMV1- F1	GGA GGA TGT TGG CAG CAT T	310	Detection	[14]
APMV1- R1	GTC AAC ATA TAC ACC TCA TC			
NDV M-F	TAGCAAATGCCTCTCCCC	1247	Sequencing	[15]
NDV M-R	GGTGGCACGCATATTAT			
NDV F-F	GGGAAGATGCAGCAGTTTG	1093		
NDV F-R	GGGTATTATTCCCAAGCC			
NDV HN-1F	GACCCTCCTGGTATCATATC	1066		
NDV HN-1R	CCCCCGATATAATCTGGG			
NDV HN-2F	GCAAAGAACACATGGCG	551		
NDV HN-2R	GAGTGATCTCTGCAACC			
NDV HN-3F	GGTTGCACTCGGATACCC	980		
NDV HN-3R	GCTTGTTCATCATCAAGC			
NDV L-F	CCCTTGCCAGGCATCAGC	825		
NDV L-R	GTTTTATCATTCTCTCTGTG			

### Total RNA extraction, cDNA synthesis, and reverse-transcription polymerase chain reaction (RT-PCR)

Total RNA was extracted from the homogenate sample using Viral Gene-spin™ Viral DNA/RNA Extraction Kit (iNtRON Biotechnology, Seoul, Korea) according to the manufacturer’s instructions. cDNA synthesis was performed using Maxime™ RT PreMix Kit (iNtRON Biotechnology) under the following conditions: 45°C for 60 min and 95°C for 5 min.

Primers, APMV1-F, and APMV1-R were used to amplify the target 310 bp of the partial F gene of NDV (Table-1), as previously described by Stäuber *et al*. [14]. Six pairs of primers were used to amplify for sequencing of the full-length F and HN genes (Table-1) as previously described by Tran *et al*. [15]. The following thermal conditions were used: An initial denaturation step at 94°C for 5 min, followed by 40 cycles at 94°C for 30 s, 52°C–58°C for 30 s (depending on the primers), and 72°C for 40 s, and a final extension step at 72°C for 10 min. A 1.2% agarose gel was used to run the PCR product. The RT-PCR product was observed under UV light.

### Nucleotide sequencing and phylogenetic analysis

The PCR products were purified using GeneClean^®^ II Kits (MP Biomedicals, Santa Ana, CA, USA). Sequencing of the F and HN genes was performed using 1^st^ BASE, Malaysia.

The obtained sequences were analyzed using GENETYX ver. 10 software (GENETYX Corp., Tokyo, Japan) and compared with other available sequences using BLAST homology searches. Deduced amino acid (AA) comparisons were performed using the Clustal W algorithm of the BioEdit (version 7.2) (https://bioedit.software.informer.com/7.2/) [16, 17]. Evolutionary distances were calculated using the aligned sequences and the Kimura 2 parameter model. Phylogenetic trees were constructed using the maximum likelihood method supported by 1000 bootstrap replicates in MEGA 6.0 software (https://www.megasoftware.net/) [18]. The nucleotide sequence obtained in this study was deposited into the GenBank database under the accession number OQ718435–OQ718441.

## Results

### Clinical and postmortem findings

**Figure-2:**
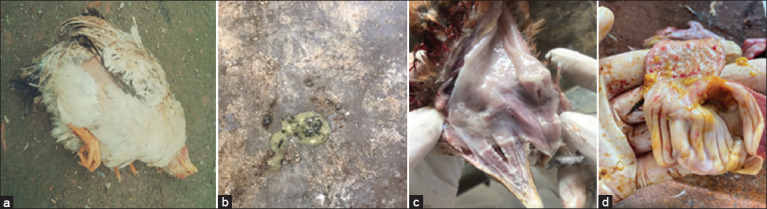
Clinical diseased chickens suspected of NDV infection: (a) Twisting of the head, (b) greenish-white diarrhea. Gross pathological lesions of NDV-suspected chickens: (c) mucus in the respiratory tract and (d) pin-point hemorrhages in the proventriculus. NDV=Newcastle disease virus.

In this study, samples were obtained from chickens with weaknesses, respiratory difficulties, depression, twisting of the head (Figure-2a), and greenish-white diarrhea (Figure-2b). The NDV-suspected chickens were examined and gross pathological changes were recorded. At necropsy, some chickens showed mucus in the respiratory tract (Figure-2c) and pin-point hemorrhages in the proventriculus (Figure-2d). The NDV-suspected chickens were collected from the farm and were raised according to the semi-grazing style, with sizes ranging from 1000 to 8000 (Table-2).

**Table-2:** Characteristics of Vietnamese Newcastle disease virus isolates analyzed in this study.

Virus strains	Year	Vaccine status	District	Farm type	Flock Size	GenBank accession no.	F-protein cleavage site (112–117)
VNUA-HN01	2021	La Sota	Hanoi	Semi-Grazing	1500	OQ718435	RRRKRF
VNUA-HN20	2022	La Sota	Hanoi	Semi-Grazing	2500	OQ718439	RRRKRF
VNUA-HP03	2022	La Sota	Haiphong	Semi-Grazing	6000	OQ718441	RRRKRF
VNUA-HP12	2022	La Sota	Haiphong	Semi-Grazing	8000	OQ718436	RRRKRF
VNUA-TN07	2022	La Sota	Thainguyen	Semi-Grazing	3000	OQ718437	RRRKRF
VNUA-TN10	2022	La Sota	Thainguyen	Semi-Grazing	1000	OQ718440	RRRKRF
VNUA-PT01	2022	La Sota	Phutho	Semi-Grazing	1000	OQ718438	RRRKRF

### Newcastle disease virus genome detection by PCR

**Figure-3:**
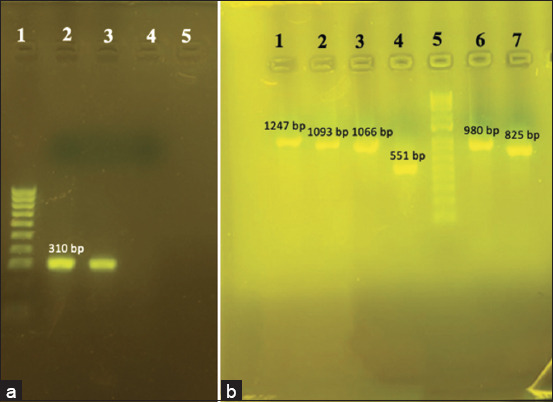
(a) Amplification of F gene fragment (310 bp) of NDV by PCR. The lanes in the photograph shows, land 1: Marker (100 bp), land 2: positive control, and land 3–5: samples; (b) amplification of F and HN gene fragments of NDV by PCR for sequencing. The lanes in the photograph shows, lane 5: Marker (1 kb), lanes 1–4, 6–7: F and HN fragments amplified by PCR using NDV M-F/R (1247 bp), NDV-F/R (1093 bp), NDV HN-1F/1R (1066 bp), NDV HN-2F/2R (551 bp), NDV HN-3F/3R (980 bp), and NDV L-F/R (825 bp), respectively. NDV=Newcastle disease virus, PCR=Reverse-transcription polymerase chain reaction, F=Fusion, HN=Hemagglutinin-neuraminidase.

Our study of NDV infection indicated that seven out of 72 (9.7%) chicken tissue samples collected from seven chicken farms in the four cities/provinces in Northern Vietnam were positive for the NDV genome by PCR method, which yielded an amplification product of 310 bp (Figure-3). In detail, two samples from HN, two samples from HP, two samples from TN, and one sample from PT were considered NDV genome positive. The isolated NDV strains were named as AAvV1/VNUA-HN01/2021 (OQ718435), AAvV1/VNUA-HN20/2022 (OQ718436), AAvV1/VNUA-HP03/2022 (OQ718441), AAvV1/VNUA-HP12/2022 (OQ718436), AAvV1/VNUA-TN07/2022 (OQ718437), AAvV1/VNUA-TN10/2022 (OQ718440), and AAvV1/VNUA-PT01/2022 with short names following VNUA-HN01, VNUA-HN20, VNUA-HP03, VNUA-HP12, VNUA-TN07, VNUA-TN10, and VNUA-PT01, respectively (Table-2).

### Analysis of predicted AA sequences encoded by F and HN genes

Full-length sequences of F and HN genes of seven current NDV strains were obtained. The AA sequence of the F-protein cleavage site for all NDV isolates was ^112^RRRKRF^117^ (Table-2). The analysis of the AA sequences encoded by the full-length F gene of the seven representative NDV strains also showed no sequence changes in all current NDV strains at the six potential N-glycosylation sites and 12 cysteine residues [19]. In addition, when compared with the consensus sequence involved in the formation of neutralizing epitopes [20], the seven NDV strains showed a K to R AA substitution at position 78 and a D to N/S AA substitution at position 170 of a stretch of AA residues 157–171 (Table-3). Furthermore, the AA residues K (at position 101) and V (at position 121) were observed in the F-protein of each current NDV strain.

The HN protein of all the current Vietnamese NDVs is composed of 571 AA. Regarding to AA residues at hemagglutinin receptor-binding sites and five N-glycosylation sites [21], the seven representative NDV strains showed the same conserved AA stretches. Regarding to the neutralizing epitopes in HN protein [22], the NDV strains in this study showed some AA substitutions. In detail, all current NDV strains contained AA substitution at position 263 (K to R). Some AA changes at the position 284 (D to Y/H), 346 (D to E), 374 (E to K), 349 (D to M), and 356 (K to I) were observed in several current NDV strains (Table-3).

**Table-3:** Amino acids involved in neutralizing epitopes of representative F and HN proteins from different other NDV strains.

Strain	F protein		HN protein						
	
	78	157–171	193–201	263	287	346	347	349	356
Consensus^a^	K	D^170^	L^193^…R^197^…H^201^	K	D	D	E	D	K
VNUA-HN01	R	N	.^b^	R	.	.	K	.	.
VNUA-HN20	R	N	I…I…N	R	Y	.	K	.	I
VNUA-HP03	R	N	.	R	H	E	.	M	.
VNUA-HP12	R	S	…….K…….	R	.	.	K	.	.
VNUA-TN07	R	N	.	R	.	.	K	.	.
VNUA-TN10	R	S	I…I…N	R	H	.	K	.	I
VNUA-PT01	R	S	.	R	.	.	.	.	.

a The consensus amino acid sequence was derived from 180 velogenic, mesogenic and lentogenic NDV strains from GenBank,

b Same as consensus amino acid sequence, F=Fusion, HN=Hemagglutinin-neuraminidase, NDV=Newcastle disease virus

### Phylogenetic characterization

**Figure-4:**
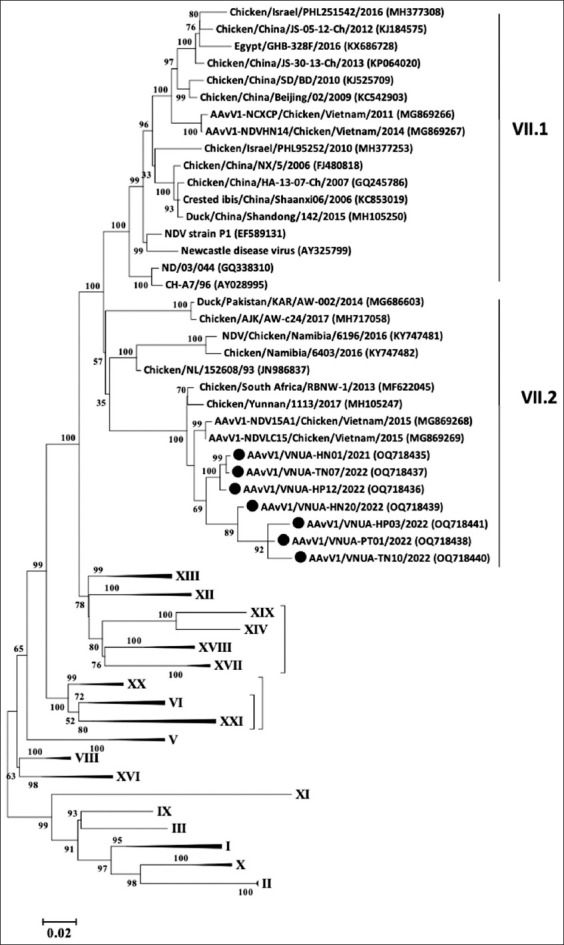
Phylogenetic tree of the completed sequence of the F gene of the current Vietnamese NDV strains compared with the sequences of other NDVs obtained from the GenBank database. The tree was constructed using the maximum likelihood method (1000 bootstrap replicates) with MEGA6 software. Bootstrap values are shown at the nodes. The sequence determined in this study is highlighted with a black circle. NDV=Newcastle disease virus.

**Figure-5:**
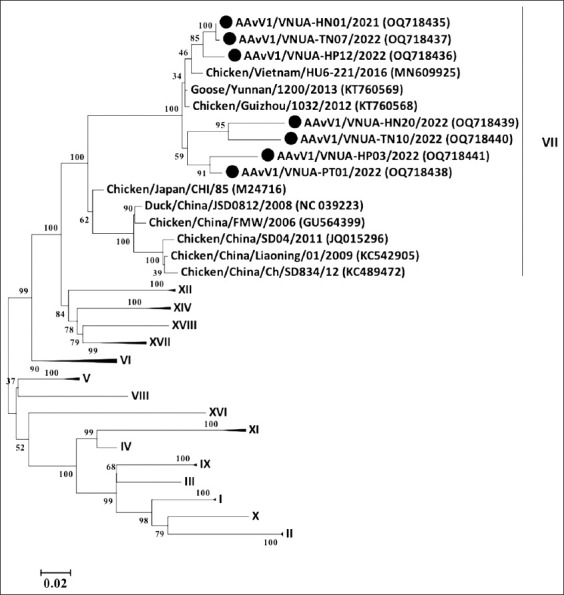
Phylogenetic tree of the completed sequence of the hemagglutinin-neuraminidase gene of the current Vietnamese NDV strains compared with the sequences of other NDVs obtained from the GenBank database. The tree was constructed using the maximum likelihood method (1000 bootstrap replicates) with MEGA6 software. Bootstrap values are shown at the nodes. The sequence determined in this study is highlighted with a black circle. NDV=Newcastle disease virus.

Phylogenetic analyses of the seven representative NDV strains using full-length F and HN genes revealed that all strains belonged to genotype VII class II and were clearly separated from the vaccine strains (genotype I and II) (Figures-4 and 5). In detail, the phylogenetic tree of the completed F gene sequence demonstrated that all current NDV strains clustered together in subgenotype VII.2 (Figure-4). In addition, the results indicated that the F gene sequences of seven NDV strains were closely related to those of the two previous Vietnamese NDV, AAvV1-NDV15A1/Chicken/Vietnam/2015 (MG869268), and AAvV1-NDVLC15/Chicken/Vietnam/2015 (MG869269), and the Chinese and Southern African NDV strains (Chicken/Yunnan/1113/2017 with Assession no. MH105247, Chicken/South Africa/RBNW-1/2013 with Assession no. MF622045, respectively).

### Evolution distances and selection profiles of the current NDV sequences

Regarding the evolutionary distances of the completed F gene sequences between the current and previous Vietnamese NDV strains, the seven NDV isolates shared a maximum of 0.22 genetic divergences from the three vaccine Vietnamese strains (NVNvacI2/2016, LSTvac/2016, and NVMvacM/2016). In addition, all current NDV strains were closely related to each other (Table-4). In particular, these NDV strains showed 0.02–0.05 genetic divergence with the two previous Vietnamese NDVs (AAvV1-NDV15A1/Chicken/Vietnam/2015 (MG869268) and AAvV1-NDVLC15/Chicken/Vietnam/2015 (MG869269) (Table-4). According to the selection profiles, eight sites in the F genes of all current NDVs were considered as a positive selection with a posterior probability of positive selection at a site equal to or more than 0.9 (Prob [α < β] ≥0.9). In addition, the analysis of selection profiles among the seven obtained NDV sequences showed 11 sites under negative selection in all current strains with a posterior probability of negative selection at a site equal to or more than 0.9 (Prob [α > β] ≥0.9) (Table-5).

**Table-4:** Comparative evolutionary distances among current and previously Vietnamese NDVs based complete fusion coding sequences.

Strain	1	2	3	4	5	6	7	8	9	10	11	12	13	14
1. VNUA-HN01		(0.00)	(0.00)	(0.00)	(0.00)	(0.01)	(0.00)	(0.01)	(0.01)	(0.00)	(0.00)	(0.02)	(0.02)	(0.02)
2. VNUA-HN20	0.03		(0.00)	(0.00)	(0.00)	(0.00)	(0.00)	(0.01)	(0.01)	(0.00)	(0.00)	(0.02)	(0.02)	(0.02)
3. VNUA-HP03	0.05	0.03		(0.00)	(0.00)	(0.00)	(0.00)	(0.01)	(0.01)	(0.00)	(0.00)	(0.02)	(0.02)	(0.02)
4. VNUA-HP12	0.01	0.03	0.04		(0.00)	(0.01)	(0.00)	(0.01)	(0.01)	(0.00)	(0.00)	(0.02)	(0.02)	(0.02)
5. VNUA-TN07	0.00	0.03	0.05	0.00		(0.01)	(0.00)	(0.01)	(0.01)	(0.00)	(0.00)	(0.02)	(0.02)	(0.02)
6. VNUA-TN10	0.06	0.03	0.02	0.06	0.06		(0.00)	(0.01)	(0.01)	(0.00)	(0.00)	(0.02)	(0.02)	(0.02)
7. VNUA-PT01	0.05	0.02	0.01	0.04	0.05	0.01		(0.01)	(0.01)	(0.00)	(0.00)	(0.02)	(0.02)	(0.02)
8. NDVHN14/2014	0.11	0.11	0.11	0.11	0.11	0.13	0.11		(0.00)	(0.01)	(0.01)	(0.02)	(0.02)	(0.02)
9. NCXCP/2011	0.11	0.11	0.11	0.11	0.11	0.13	0.11	0.00		(0.01)	(0.01)	(0.02)	(0.02)	(0.02)
10. NDV15A1/2015	0.03	0.03	0.04	0.02	0.03	0.05	0.04	0.09	0.09		(0.00)	(0.02)	(0.02)	(0.02)
11. NDVLC15/2015	0.02	0.03	0.04	0.02	0.02	0.05	0.04	0.09	0.09	0.00		(0.02)	(0.02)	(0.02)
12. NVNvacI2/2016	0.18	0.18	0.18	0.17	0.18	0.19	0.18	0.16	0.16	0.16	0.16		(0.01)	(0.01)
13. LSTvac/2016	0.19	0.2	0.2	0.19	0.19	0.22	0.20	0.18	0.16	0.18	0.18	0.13		(0.00)
14. NVMvacM/2016	0.18	0.19	0.2	0.18	0.18	0.22	0.20	0.18	0.18	0.18	0.18	0.13	0.00	

The number of base substitutions per site is shown by averaging overall sequence pairs between different groups. Standard error estimate is shown above the diagonal (in parentheses) and was obtained by a bootstrap procedure (1,000 replicates). Divergences between the vaccine strains and isolates are underlined. The analysis involved 14 nucleotide sequences. Codon positions included were 1^st^+2^nd^+3^rd^+non-coding. All positions containing gaps and missing data were eliminated. There were a total of 1653 positions in the final data. Evolutionary analyses were conducted in MEGA6, NDV=Newcastle disease virus

**Table-5:** Substituted amino acid positions as negative selection in the F gene sequences of the current Vietnamese NDV strains.

Amino acid position	α	β	β-α	Prob [α > β]	Prob [α < β]
192	2.24	19.84	17.60	0.04	0.92
193	1.65	15.51	13.86	0.05	0.92
254	24.89	0.76	−24.12	0.98	0.00
272	2.01	20.07	18.06	0.04	0.93
278	24.57	0.87	−23.69	0.98	0.00
288	15.23	0.79	−14.44	0.90	0.07
292	25.51	0.79	−24.71	0.98	0.00
409	25.79	0.89	−24.9	0.98	0.00
412	2.50	20.09	17.58	0.04	0.91
424	29.70	0.82	−28.88	0.99	0.00
430	29.69	0.81	−28.88	0.99	0.00
504	29.02	0.96	−28.05	0.99	0.00
517	24.85	0.84	−24.01	0.98	0.00
524	1.93	14.60	12.66	0.06	0.95
525	17.70	1.02	−16.68	0.96	0.01
539	38.57	0.97	−37.60	0.99	0.00
546	1.92	19.73	17.81	0.04	0.93
549	1.54	14.07	12.53	0.05	0.91
551	1.86	14.08	12.21	0.06	0.90

α=Indicates posterior synonymous substitution rate at a site, β=Indicates posterior non-synonymous substitution rate at a site, α > β=Negative selection, α < β=Positive selection; α=β=Neutral selection, Prob [α > β] ≥ 0.9=Posterior probability of negative selection at a site, Prob[α < β] ≥ 0.9=Posterior probability of positive selection at a site, F=Fusion, NDV=Newcastle disease virus

## Discussion

Even though intensive ND vaccination programs have been applied, NDV remains a serious threat to the poultry industry worldwide. Genotype VII class II has been portrayed as the predominant genotype that caused ND outbreaks among vaccinated commercial flocks [23]. In Vietnam, genotype VII was reported as a virulent strain causing ND outbreaks in domestic chickens between 2007 and 2015 [11, 12]. In this study, we detected seven NDV strains belonging to subgenotype VII.2 class II obtained from ND-vaccinated chicken farms in the north of Vietnam from 2021 to 2022. It appears to be confirmed that genotype VII class II is still a circulating NDV genotype in the poultry industry in Vietnam.

The current study reported that NDV has distinctive clinical signs such as respiratory difficulties, depression, head twisting, and greenish-white diarrhea. This is consistent with the results of previous studies by Alexander [24], Bereket *et al*. [25], and Khorajiya *et al*. [26]. A previous study by Wise *et al*. [27] reported that NDV-infected chickens also showed enlargement and inflammation of the eyes, diarrhea, and lack of appetite. Indeed, diarrhea was observed in the sick chickens obtained in the study. Other clinical signs were reported differently in the affected host organs. At necropsy, the mucus in the respiratory tract and pin-point hemorrhages in the proventriculus were observed in the suspected chickens. These results strongly agree with those of other studies [25, 28]. Based on the clinical signs and the postmortem findings, the chickens obtained in the study were considered to be infected with NDV.

In addition, the PCR results confirmed the presence of the NDV genome in the seven obtained samples. It is noted that all chicken flocks were vaccinated using La Sota strain; however, the ND outbreaks still occur [29]. Unfortunately, vaccination procedures have not been obtained in detail for inclusion in this report. Newcastle disease virus genotype VII.2 is reported to be responsible for the fifth NDV panzootic and continuously spread in the poultry industry [30]. In Vietnam, genotype VII.2 was recently reported in chickens in 2015 [11]. In the present study, the results of the phylogenetic analysis of the full-length F and HN gene sequences also indicated that the NDV of subgenotype VII.2 class II has been continuously circulated among the broiler chicken farms in the North of Vietnam from 2021 to 2022. The diversity and the presence of multiple NDV sub-genotypes have been noted in many European and Asian countries. In addition, genotype VII has expanded its endemicity to Central Africa. It is suggested that genotype VII NDVs have been widespread in nature, temporal and spatial predominance worldwide. Therefore, the information on NDV molecular characterization obtained from vaccinated chicken farms appears to be useful for the improvement of ND control measures.

In this study, all seven current NDV isolates were classified as virulent based on the AA sequence of the F0 protein proteolytic cleavage site (^112^RRRKRF^117^). Furthermore, the phenylalanine residue at position 117 of the F-protein and the 571 AA in the length of the HN protein suggested that the seven representative isolates had characteristic features of the virulent NDV strains in the study [31–33]. This finding was strong agreement with some reports that ND outbreaks were caused by virulent genotype VII NDV in vaccinated flocks in several countries such as India [34], China [35], Pakistan [36], and Bangladesh [37]. It is necessary to clarify why the number of ND outbreaks is continuously increasing in vaccinated chicken flocks.

Obtaining additional completed F and HN gene sequences from the field samples is necessary to understand well about the diversity of the NDV genome in Vietnam. In this study, the full-length F and HN gene sequences of seven genotype VII.2 isolates were evaluated and characterized. Compared to the NDV consensus sequence, the seven current NDV strains had some AA substitutions in the neutralizing epitopes of the F and HN proteins, which were the targets of neutralizing antibodies (Table-3). Notably, the AA substitutions at residue 78 in the F-protein of these current NDVs were considered to affect the activity of neutralizing antibodies. F-protein AA substitutions at 78 and 79 have been reported to alter the antigenicity of NDV [35, 38, 39]. In addition, the AA residues at position 347 in the HN protein may influence the antigenic variation of NDV [40]. Furthermore, the previous study by Umali *et al*. [41] have reported that NDV strains containing the substitution residues at position 347 in the HN protein showed 2-to 3-fold reductions in neutralizing antibody titers compared with those without this substitution. However, whether these mutations observed in the current NDV strains were a reason for outbreaks in the vaccinated chickens or not is still unclear well. In addition, the current study clearly showed that Vietnamese NDV isolates shared distance from the NDV vaccine strains (genotype I and II) based on phylogenetic analysis. This is in line with the results of the previous study by Dimitrov *et al*. [42], which reported a genetically distant (18.3–26.6% nucleotide distance) between the virulent and the genotype I and II NDV strains. To understand how the virulent NDVs infect vaccinated chickens, the identified mutation might provide insight into the evolution of NDVs in the future.

The evolutionary selection profiles of the Vietnamese field strains obtained in this study showed 11 sites under negative selection and eight sites under positive selection. The result of previous studies by Bush [43], and Kosiol *et al*. [44] indicated that positive selection sites might lead to an increase in genetic variation. This finding may be useful for understanding well the dynamic of NDV infection in vaccinated chickens based on future site-directed mutagenesis.

## Conclusion

The investigation reported all field isolates from vaccinated commercial farms with virulent characteristics belonging to the subgenotype VII.2 in the North of Vietnam from 2021 to 2022. The generated data provide a comprehensive molecular characterization of NDVs circulating in Northern Vietnam. This present finding will also facilitate future studies of the evolution of NDVs, particularly highlighting the importance of molecular characterization in NDVs.

## Authors’ Contributions

THGT: Designed the research, collected samples, analyzed data, and wrote the manuscript. VHD and TMLH: Designed the research, analyzed data, and edited the manuscript. VTL, TNV, and HAD: Collected samples and edited the manuscript. All authors have read, reviewed, and approved the final manuscript.

## References

[1] Walker P.J, Siddell S.G, Lefkowitz E.G, Mushegian A.R, Adriaenssens E.M, Alfenas-Zerbini P, Dempsey D.M, Dutilh B.E, García M.L, Hendrickson R.C, Junglen S, Krupovic M, Kuhn J.H, Lambert A.J, Łobocka M, Oksanen H.M, Orton R.J, Robertson D.L, Rubino L, Sabanadzovic S, Simmonds P, Smith D.P, Suzuki N, Van Doorslaer K, Vandamme A.M, Varsani A, Zerbini F.M (2022). Recent changes to virus taxonomy ratified by the International Committee on Taxonomy of Viruses (2022). Arch. Virol.

[2] Dimitrov K.M, Abolnik C, Afonso C.L, Albina E, Bahl J, Berg M, Briand F.X, Brown I.H, Choi K.S, Chvala I, Diel D.G, Durr P.A, Ferreira H.L, Fusaro A, Gil P, Goujgoulova G.V, Grund C, Hicks J.T, Joannis T.M, Torchetti M.K, Kolosov S, Lambrecht B, Lewis N.S, Liu H, Liu H, McCullough S, Miller P.J, Monne I, Muller I.P, Munir M, Reischak D, Sabra M, Samal S.K, de Almeida R.S, Shittu I, Snoeck C.J, Suarez D.L, Van Borm S, Wang Z, Wong F.Y.K (2019). Updated unified phylogenetic classification system and revised nomenclature for Newcastle disease virus. Infect. Genet Evol.

[3] Welch C.N, Shittu I, Abolnik C, Solomon P, Dimitrov K.M, Taylor T.L, Williams-Coplin D, Goraichuk I.V, Meseko C.A, Ibu J.O, Gado D.A, Joannis T.M, Afonso C.L (2019). Genomic comparison of Newcastle disease viruses isolated in Nigeria between 2002 and 2015 reveals circulation of highly diverse genotypes and spillover into wild birds. Arch. Virol.

[4] Suarez D.L, Miller P.J, Koch G, Mundt E, Rautenschlein S, Swayne D.E (2020). Newcastle disease, other avian paramyxoviruses, and avian metapneumovirus infections. Diseases of Poultry.

[5] Gaurav S, Deka P, Das S, Deka P, Hazarika R, Kakati P, Kumar A, Kumar S (2022). Isolation of genotype VII avian orthoavulavirus serotype 1 from barn owl from Northeast India. Avian Pathol.

[6] Saputri M.E, Poetri O.N, Soejoedono R.D (2021). Phylogenetic studies of Newcastle disease virus isolated from poultry flocks in South Sulawesi Province, Indonesia, in 2019. J. Adv. Vet. Anim. Res.

[7] Twabela A.T, Nguyen L.T, Masumu J, Mpoyo P, Mpiana S, Sumbu U.J, Okamatsu M, Matsuno K, Isoda N, Zecchin B, Monne I, Sakoda Y (2021). A new variant among Newcastle disease viruses isolated in the Democratic Republic of the Congo in 2018 and 2019. Viruses.

[8] Steensels M, Van Borm S, Mertens I, Houdart P, Rauw F, Roupie V, Snoeck C.J, Bourg M, Losch S, Beerens N, van den Berg T, Lambrecht B (2021). Molecular and virological characterization of the first poultry outbreaks of Genotype VII.2 velogenic avian orthoavulavirus Type 1 (NDV) in North-West Europe, BeNeLux, 2018. Transbound. Emerg. Dis.

[9] Kgotlele T, Modise B, Nyange J.F, Thanda C, Cattoli G, Dundon W.G (2020). First molecular characterization of avian paramyxovirus-1 (Newcastle disease virus) in Botswana. Virus Genes.

[10] Tu T.D, Phuc K.V, Dinh N.T, Quoc D.N, Spradbrow P.B (1998). Vietnamese trials with a thermostable Newcastle disease vaccine (strain I2) in experimental and village chickens. Prev. Vet. Med.

[11] Le X.T.K, Doan H.T.T, Le T.H (2018). Molecular analysis of Newcastle disease virus isolates reveals a novel XIId subgenotype in Vietnam. Arch. Virol.

[12] Choi K.S, Kye S.J, Kim J.Y, To T.L, Nguyen D.T, Lee Y.J, Choi J.G, Kang H.M, Kim K.I, Song B.M, Lee H.S (2014). Molecular epidemiology of Newcastle disease viruses in Vietnam. Trop. Anim. Health Prod.

[13] Susta L, Miller P.J, Afonso C.L, Brown C.C (2011). Clinicopathological characterization in poultry of three strains of Newcastle disease virus isolated from recent outbreaks. Vet. Pathol.

[14] Stäuber N, Brechtbühl K, Bruckner L, Hofmann M.A (1995). Detection of Newcastle disease virus in poultry vaccines using the polymerase chain reaction and direct sequencing of amplified cDNA. Vaccine.

[15] Tran G.T.H, Mananggit M.R, Abao L.N.B, Van Dong H, Takeda Y, Ogawa H, Imai K (2021). Molecular characterization of a Newcastle disease virus isolate from a diseased chicken in the Philippines in 2017. Jpn. J. Vet. Res.

[16] Hall T.A (1999). BioEdit:A user-friendly biological sequence alignment editor and analysis program for Windows 95/98/NT. Nucleic Acids Symp. Ser.

[17] Thompson J.D, Higgins D.G, Gibson T.J (1994). CLUSTAL W:Improving the sensitivity of progressive multiple sequence alignment through sequence weighting, position-specific gap penalties and weight matrix choice. Nucleic Acids Res.

[18] Tamura K, Stecher G, Peterson D, Filipski A, Kumar S (2013). MEGA6:Molecular evolutionary genetics analysis version 6.0. Mol. Biol. Evol.

[19] White J.M, Delos S.E, Brecher M, Schornberg K (2008). Structures and mechanisms of viral membrane fusion proteins:Multiple variations on a common theme. Crit. Rev. Biochem. Mol. Biol.

[20] Umali D.V, Ito H, Suzuki T, Shirota K, Katoh H, Ito T (2013). Molecular epidemiology of Newcastle disease virus isolates from vaccinated commercial poultry farms in non-epidemic areas of Japan. Virol. J.

[21] Connaris H, Takimoto T, Russell R, Crennell S, Moustafa I, Portner A, Taylor G (2002). Probing the sialic acid binding site of the hemagglutinin-neuraminidase of Newcastle disease virus:Identification of key amino acids involved in cell binding, catalysis, and fusion. J. Virol.

[22] Kelley L.A, Mezulis S, Yates C.M, Wass M.N, Sternberg M.J.E (2015). The Phyre2 web portal for protein modeling, prediction and analysis. Nat. Protoc.

[23] Rtishchev A, Treshchalina A, Shustova E, Boravleva E, Gambaryan A (2023). An outbreak of Newcastle disease virus in the Moscow region in the summer of 2022. Vet. Sci.

[24] Alexander D.J (2000). Newcastle disease and other avian paramyxoviruses. Rev. Sci. Tech.

[25] Bereket M, Beilul G, Fitsum N, Yodahi P, Yohana xS (2017). Outbreak investigation of Newcastle disease virus from vaccinated chickens in Eritrea. Afr. J. Biotechnol.

[26] Khorajiya J.H, Pandey S, Ghodasara P.D, Joshi B.P, Prajapati K.S, Ghodasara D.J, Mathakiya R.A (2015). Patho-epidemiological study on Genotype-XIII Newcastle disease virus infection in commercial vaccinated layer farms. Vet. World.

[27] Wise M.G, Suarez D.L, Seal B.S, Pedersen J.C, Senne D.A, King D.J, Kapczynski D.R, Spackman E (2004). Development of a real-time reverse-transcription PCR for detection of Newcastle disease virus RNA in clinical samples. J. Clin. Microbiol.

[28] Ashraf A, Shah M.S.D, Habib M, Hussain M, Mahboob S, Al-Ghanim K (2016). Isolation, identification and molecular characterization of highly pathogenic Newcastle disease virus from field outbreaks. Braz. Arch. Biol. Technol.

[29] Hu Z, He X, Deng J, Hu J, Liu X (2022). Current situation and future direction of Newcastle disease vaccines. Vet. Res.

[30] Miller P.J, Haddas R, Simanov L, Lublin A, Rehmani S.F, Wajid A, Bibi T, Khan T.A, Yaqub T, Setiyaningsih S, Afonso C.L (2015). Identification of new sub-genotypes of virulent Newcastle disease virus with potential panzootic features. Infect. Genet. Evol.

[31] Collins M.S, Bashiruddin J.B, Alexander D.J (1993). Deduced amino acid sequences at the fusion protein cleavage site of Newcastle disease viruses showing variation in antigenicity and pathogenicity. Arch. Virol.

[32] OIE (2022). Newcastle disease (Infection with Newcastle disease virus). OIE Terrestrial Manual 2022:Manual of Diagnostic Tests and Vaccines for Terrestrial Animals. Ch. 3.3.14.

[33] Römer-Oberdörfer A, Werner O, Veits J, Mebatsion T, Mettenleiter T.C (2003). Contribution of the length of the HN protein and the sequence of the F-protein cleavage site to Newcastle disease virus pathogenicity. J. Gen. Virol.

[34] Mariappan A.K, Munusamy P, Kumar D, Latheef S.K, Singh S.D, Singh R, Dhama K (2018). Pathological and molecular investigation of velogenic viscerotropic Newcastle disease outbreak in a vaccinated chicken flocks. Virusdisease.

[35] Qin Z.M, Tan L.T, Xu H.Y, Ma B.C, Wang Y.L, Yuan X.Y, Liu W.J (2008). Pathotypical characterization and molecular epidemiology of Newcastle disease virus isolates from different hosts in China from 1996 to 2005. J. Clin. Microbiol.

[36] Rehmani S.F, Wajid A, Bibi T, Nazir B, Mukhtar N, Hussain A, Lone N.A, Yaqub T, Afonso C.L (2015). Presence of virulent Newcastle disease virus in vaccinated chickens in farms in Pakistan. J. Clin. Microbiol.

[37] Nooruzzaman M, Hossain I, Begum J.A, Moula M, Khaled S.A, Parvin R, Chowdhury E.H, Islam M.R, Diel D.G, Dimitrov K.M (2022). The first report of a virulent Newcastle disease virus of genotype VII.2 causing outbreaks in chickens in Bangladesh. Viruses.

[38] Ke G.M, Yu S.W, Ho C.H, Chu P.Y, Ke L.Y, Lin K.H, Tsai Y.C, Liu H.J, Lin M.Y (2010). Characterization of newly emerging Newcastle disease viruses isolated during 2002–2008 in Taiwan. Virus Res.

[39] Xu Q, Sun J, Gao M, Zhao S, Liu H, Zhang T, Han Z, Kong X, Liu S (2017). Genetic, antigenic, and pathogenic characteristics of Newcastle disease viruses isolated from geese in China. J. Vet. Diagn. Invest.

[40] Cho S.H, Kwon H.J, Kim T.E, Kim J.H, Yoo H.S, Kim S.J (2008). Variation of a Newcastle disease virus hemagglutinin-neuraminidase linear epitope. J. Clin. Microbiol.

[41] Umali D.V, Ito H, Shirota K, Katoh H, Ito T (2014). Characterization of complete genome sequence of genotype VI and VII velogenic Newcastle disease virus from Japan. Virus Genes.

[42] Dimitrov K.M, Afonso C.L, Yu Q, Miller P.J (2017). Newcastle disease vaccines-A solved problem or a continuous challenge?. Vet. Microbiol.

[43] Bush R.M (2001). Predicting adaptive evolution. Nat. Rev. Genet.

[44] Kosiol C, Bofkin L, Whelan S (2006). Phylogenetics by likelihood:Evolutionary modeling as a tool for understanding the genome. J. Biomed. Inform.

